# Reducing Salt Intake in China with “Action on Salt China” (ASC): Protocol for Campaigns and Randomized Controlled Trials

**DOI:** 10.2196/15933

**Published:** 2020-04-09

**Authors:** Puhong Zhang, Feng J He, Yuan Li, Changning Li, Jing Wu, Jixiang Ma, Bing Zhang, Huijun Wang, Yinghua Li, Junhua Han, Rong Luo, Jing He, Xian Li, Yu Liu, Changqiong Wang, Monique Tan, Graham A MacGregor, Xinhua Li

**Affiliations:** 1 The George Institute for Global Health at Peking University Health Science Center Beijing China; 2 Faculty of Medicine University of New South Wales Sydney Australia; 3 Wolfson Institute of Preventive Medicine, Barts and The London School of Medicine & Dentistry Queen Mary University of London London United Kingdom; 4 Surveillance Department Chinese Center for Health Education Beijing China; 5 The National Center for Chronic and Noncommunicable Disease Control and Prevention The Chinese Center for Disease Control and Prevention Beijing China; 6 Chronic Diseases and Aging Health Management Division The Chinese Center for Disease Control and Prevention Beijing China; 7 National Institute for Nutrition and Health The Chinese Center for Disease Control and Prevention Beijing China; 8 Food Policy China National Center for Food Safety Risk Assessment Beijing China; 9 School of Computing Beihang University Beijing China; 10 Chinese Center for Disease Control and Prevention Beijing China

**Keywords:** sodium, dietary, 24-hour urinary sodium, salt reduction, randomized controlled trials, scaling-up, China

## Abstract

**Background:**

Salt intake in China is over twice the maximum recommendation of the World Health Organization. Unlike most developed countries where salt intake is mainly derived from prepackaged foods, around 80% of the salt consumed in China is added during cooking.

**Objective:**

Action on Salt China (ASC), initiated in 2017, aims to develop, implement, and evaluate a comprehensive and tailored salt reduction program for national scaling-up.

**Methods:**

ASC consists of six programs working in synergy to increase salt awareness and to reduce the amount of salt used during cooking at home and in restaurants, as well as in processed foods. Since September 2018, two health campaigns on health education and processed foods have respectively started, in parallel with four open-label cluster randomized controlled trials (RCTs) in six provinces across China: (1) app-based intervention study (AIS), in which a mobile app is used to achieve and sustain salt reduction in school children and their families; (2) home cook-based intervention study (HIS), in which family cooks receive support in using less salt; (3) restaurant-based intervention study (RIS) targeting restaurant consumers, cooks, and managers; and (4) comprehensive intervention study (CIS), which is a real-world implementation and evaluation of all available interventions in the three other RCTs. To explore the barriers, facilitators, and effectiveness of delivering a comprehensive salt reduction intervention, these RCTs will last for 1 year (stage 1), followed by nationwide implementation (stage 2). In AIS, HIS, and CIS, the primary outcome of salt reduction will be evaluated by 24-hour urinary sodium excretion in 6030 participants, including 5436 adults and 594 school children around 8-9 years old. In RIS, the salt content of meals will be measured by laboratory food analysis of the 5 best-selling dishes from 192 restaurants. Secondary outcomes will include process evaluation; changes in knowledge, attitude, and practice on salt intake; and economic evaluation.

**Results:**

All RCTs have been approved by Queen Mary Research Ethics Committee and the Institutional Review Boards of leading institutes in China. The research started in June 2017 and is expected to be completed around March 2021. The baseline investigations of the four RCTs were completed in May 2019.

**Conclusions:**

The ASC project is progressing smoothly. The intervention packages and tailored components will be promoted for salt reduction in China, and could be adopted by other countries.

**Trial Registration:**

Chinese Clinical Trial Registry. AIS: ChiCTR1800017553; https://tinyurl.com/vdr8rpr. HIS: ChiCTR1800016804; https://tinyurl.com/w8c7x3w. RIS: ChiCTR1800019694; https://tinyurl.com/uqkjgfw. CIS: ChiCTR1800018119; https://tinyurl.com/s3ajldw.

**International Registered Report Identifier (IRRID):**

DERR1-10.2196/15933

## Introduction

Humans only require a very small amount of salt (ie, around 1 g/day) to maintain physiological function [1-3]. High salt intake is the major cause of raised blood pressure [4] and is the leading risk factor of total death and disability-adjusted life years in China [5]. Compelling evidence has shown that a lower salt intake is associated with a reduced risk of cardiovascular disease (CVD) and total mortality [6,7]. Salt reduction is one of the most cost-effective measures to prevent hypertension and CVD [4,8]. The World Health Organization (WHO) has recommended a 30% reduction in population salt intake by 2025, and also set a target of <5 g/day for all adults with even lower levels for children [1]. Accordingly, many developed countries have started salt reduction initiatives [9]. Salt intake has been successfully reduced in Finland and the United Kingdom, accompanied by falls in population blood pressure and CVD-related mortality [10]; however, developing countries are lagging behind.

China is the largest developing country, accounting for one fifth of the global population. Salt intake in China is among the highest in the world, with adults consuming on average above 10 g/day [11,12], which is more than twice the WHO recommended limit [1]. Approximately 80% of the salt in the Chinese diet is added by consumers during cooking [13]. With rapid and sustained urbanization, the amount of salt intake from restaurants and prepackaged foods is also increasing. A recent study showed that the major sources of salt intake for urban adults of working age are home cooking (50.1%, 40.8% of which is derived from cooking salt and 9.3% from various condiments), food prepared by restaurants (43.3%), and prepackaged food (6.6%) [14].

In most developed countries, where 80% of salt intake is derived from prepackaged food, the major strategy of salt reduction is to set salt targets; that is, to encourage food producers to gradually reduce the amount of salt used in their food products [15]. However, salt reduction is more challenging in China and many other developing countries owing to the difficulty in changing individuals’ dietary behavior. It is of paramount importance to develop strategies and specific solutions to improve: (1) the environment, so that it encourages and facilitates salt reduction; (2) consumer knowledge, attitude, and practice (KAP) of eating food with reduced salt content; (3) family or restaurant cooks’ knowledge and skills in reducing salt use during cooking; and (4) motivation of the food industry to reduce salt use in processed foods.

The central government of China has set a target of a 20% reduction in mean population salt intake by 2030 as one of the key components of China’s health development agenda “Healthy China 2030” [16]. The government-led initiative “Healthy Lifestyle for All” has also identified salt reduction as one of the most important strategies to prevent noncommunicable diseases. Responding to the national call for salt reduction, several regional salt reduction projects have been undertaken in various regions of China as part of routine work in disease control and prevention systems. However, none of these existing programs has been properly evaluated for effectiveness and sustainability, and it is not known whether they can be rolled out across the whole country.

To overcome the above challenges, a collaboration unit called “Action on Salt China” (ASC) was established in June 2017, funded by the UK National Institute for Health Research (NIHR). ASC was built upon an existing collaboration between Queen Mary University of London in the United Kingdom, and The George Institute for Global Health (TGI) in China, as well as previous research and implementation experience on salt reduction [17]. In addition, ASC has included almost all of the key national organizations related to salt reduction, including the Chinese Center for Disease Control and Prevention (China CDC), Chinese Center for Health Education (CCHE), and China National Center for Food Safety Risk Assessment (CFSA), as well as local health and education authorities. The program aims to design several standardized, effective, and sustainable salt reduction packages targeting the major challenges in salt reduction in China, and to scale them up after appropriate evaluation.

ASC is running two national health campaigns and four randomized controlled trials (RCTs) testing interventions on major sources of salt intake. Although the protocol and results of each RCT will be published separately, it is worthwhile to report the overall design of ASC so as to improve public understanding of its rationale and design as a whole. With this aim, this paper introduces ASC’s overall goals and strategies, governance, proposed solutions to main challenges in salt reduction, uniformed design of intervention packages and evaluations, plan for scaling-up, as well as other ancillary work.

## Methods

### Goals and Objectives

The goal of ASC is to reduce salt intake by 15% by 2021 in the six target provinces in China. The specific objectives are to reduce salt intake by at least 1 g/day (about 17 mmol/day) at home, and to reduce salt use by at least 0.5 g (about 8.5 mmol) per 100 g in restaurant dishes. To reach these goals, several programs targeting the major sources of salt intake in China have been developed and are being implemented.

### Strategy and Overall Design

To achieve these goals and objectives, the ASC project developed six programs targeting the low health literacy related to salt reduction and the three sources of salt intake in China: home cooking, restaurant foods, and prepackaged foods. Program 1 is a salt reduction education campaign, which will form the basis of all the other programs. Program 2, app-based intervention study (AIS), is a primary school-based program delivering salt reduction activities for the homes of school children, based on the assumption that parents and grandparents are more likely to change their habit of high salt intake for the sake of the health of their children and grandchildren [18,19]. Program 3, home cook-based intervention study (HIS), will establish a community-level training and support system to help family cooks reduce salt use in home cooking. Program 4, restaurant-based intervention study (RIS), aims to create an environment in restaurants that is conducive to consumers opting for reduced-salt dishes, and to train cooks to use less salt while cooking. Program 5, comprehensive intervention study (CIS), simulates the real-world implementation of all of the intervention packages or components developed in Programs 1-4 with the purpose of identifying barriers and facilitators when scaling up. Program 6 is a two-fold campaign with the aims of educating and supporting consumers choosing less salted prepackaged foods (Program 6.1), and convincing food manufacturers to reformulate their food products by gradually reducing the amount of salt added (Program 6.2).

### Governance of the Action on Salt China Unit and Programs

**Figure 1:**
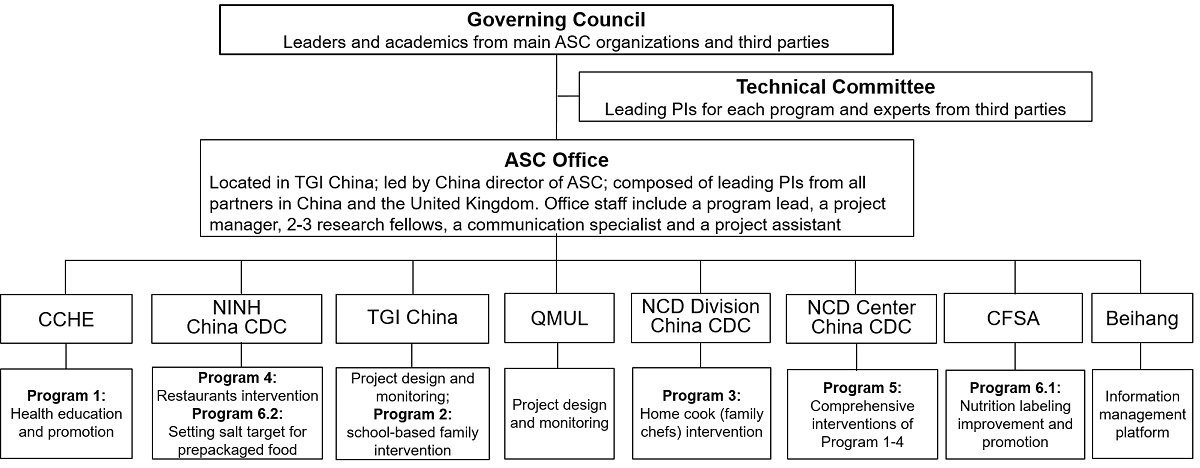
Governance and specific programs of Action on Salt China (ASC) program. PI: principal investigator; China CDC: China Centers for Disease Control; CCHE: Chinese Center for Health Education; NINH: National Institute for Nutrition and Health, China CDC; TGI China: The George Institute for Global Health China; QMUL: Queen Mary University of London; NCD Division: Division of NCD Control and Community Health, China CDC; NCD Center: National Center for Chronic and Non-communicable Disease Control and Prevention, China CDC; CFSA: China National Center for Food Safety Risk Assessment; Beihang: Beihang University (Or Beijing University of Aeronautics and Astronautics).

The governance and management structure of the ASC unit and programs are illustrated in Figure 1. The governing council consists of 10 members, 5 of whom are the top leaders from the 5 member organizations of the ASC unit. The other 5 members are academics from independent organizations, including the National Preventive Medicine Association (2 members with Chairman), China Nutrition Society, Chinese Hypertension League, and Peking University Health Science Center. The governing council provides guidance by (1) reviewing ASC’s goals and strategies, (2) setting the tone for cooperation and communication, and (3) evaluating ASC’s overall performance and achievement every year. The technical committee is composed of the leading principal investigators of all programs and independent experts in salt reduction. The committee members will work together to finalize documents (eg, research protocols and education materials), provide support in program implementation, and act as the ultimate decision maker in handling any practical issues. Supervised and supported by the governing council and technical committee, the ASC office, located in TGI China, will be responsible for coordinating all of the partners to implement the proposed programs, and to ensure the smooth running of the whole unit and its programs to achieve quality outputs in a timely manner.

### Program Implementation

A two-stage strategy is adopted to ensure that the proposed salt reduction programs are well prepared, evaluated, and tailored. At stage 1 (first 2 years), the intervention packages in each program will be developed and evaluated, before being scaled-up and integrated as policies at stage 2.

At stage 1, the different intervention packages of Programs 2-5 will be evaluated with open-label cluster RCTs in various settings. The four RCTs will be conducted collaboratively under the same governance and time frame. Evaluation will be performed using standardized questionnaires, physical examinations, laboratory tests, and data collection systems that are as identical as possible so that the data collected across the RCTs can be pooled together for overall salt intake estimation and overall effectiveness evaluation.

The key features of the six programs of ASC at stage 1, including the theories and key intervention components of the four RCTs (Programs 2-5 at stage 1), are summarized in Table 1. Separate protocols for each of the four RCTs describing the study setting and participants, randomization, intervention, sample size calculation, outcomes, and data collection and analysis will be published before the end of stage 1. These results (especially those related to effectiveness and process evaluation) will also be published separately after the RCTs are completed.

The study participants are grade 3 primary school students (8-9 years old) and their parents/grandparents (1 student and 2 adults for each family) in AIS, home cooks and their family members (1 home cook and one other adult member for each family) in HIS, and adults (1 adult from each participating family) in CIS. Although the participant recruitment may vary among AIS, HIS, and CIS, in all cases, these will be local residents with no plan to move out of the city or village within 24 months, and agree to participate in the studies. The exclusion criteria are (1) pregnant women and those actively lactating; (2) individuals who are currently participating in any other clinical trials; (3) those with severe psychiatric or physical diseases that might impact intervention and follow-up; (4) individuals who are unable or not suitable to collect 24-hour urine due to the following conditions: aconuresis; acute/chronic urinary tract infection, vaginal infection and perianal infection; acute hemorrhagic diseases in the urinary tract, vagina, and digestive tract; and severe vomiting and diarrheic symptoms. In RIS, the study subjects are restaurants with dish salt content as the primary outcome, which will be evaluated using the average sodium content of the 5 best-selling dishes.

At the end of stage 1, the education materials and the effective intervention packages or components will be combined as a scale-up intervention package on salt reduction (SIPS) for broad use at stage 2. A final evaluation to assess the impact of the scaling-up and the lasting effectiveness of the intervention packages will be carried out at the end of 1 year of scale-up. The SIPS will subsequently be further promoted over a larger scale across China using existing platforms and resources such as China’s Healthy Lifestyle for All Initiative of the China CDC and the Chinese Center for Health Education.

**Figure 2:**
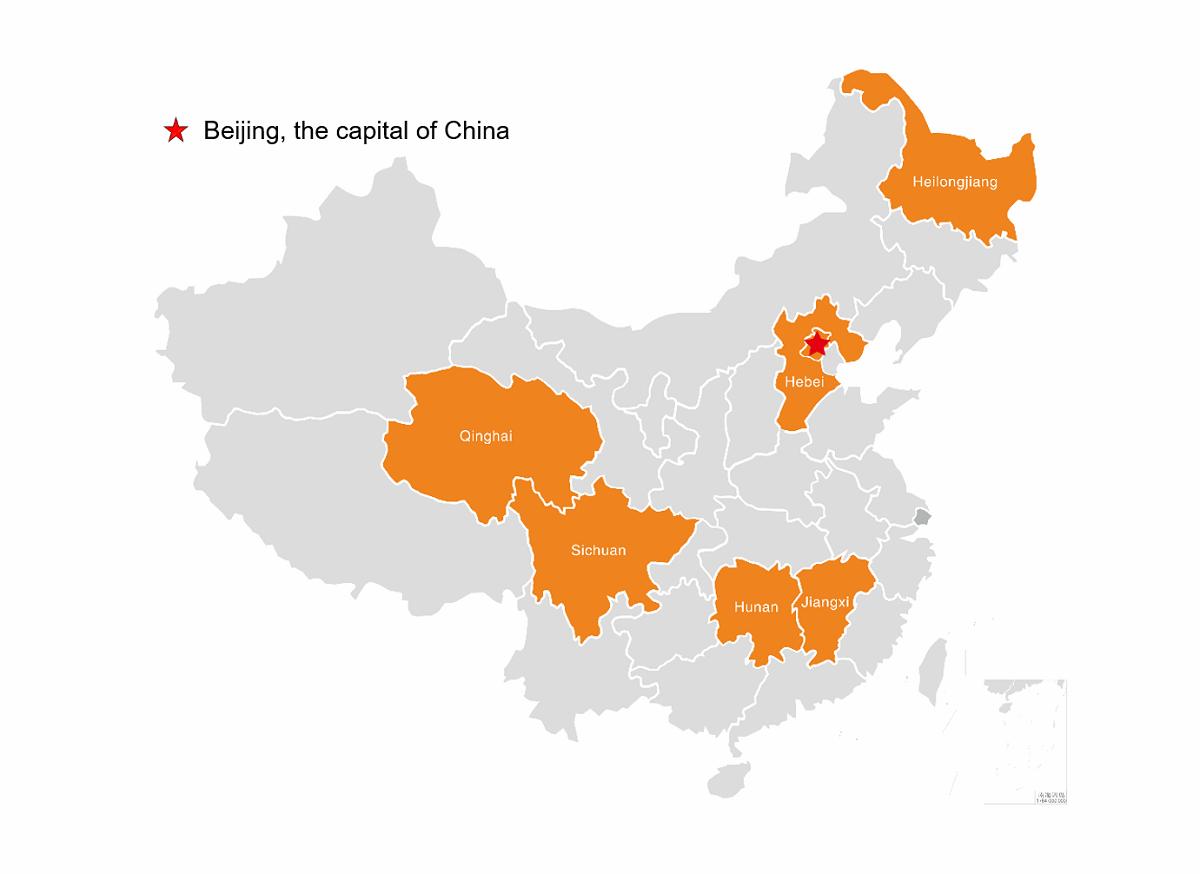
The study sites of the Action on Salt China (ASC) cluster of randomized controlled trials.

To avoid contamination in control groups, all RCTs are being conducted in different counties or districts of the six provinces: Heilongjiang, Hebei, Hunan, Jiangxi, Sichuan, and Qinghai, which cover the north, south, central, east, and west part of China (Figure 2). HIS, RIS, and CIS are carried out in the above six provinces, whereas AIS is conducted in only three provinces: Hebei (north), Sichuan (central), and Hunan (south).

To facilitate implementation, ASCloud, a cloud-based information system, has been designed and developed by Beihang University to support health education and promotion to the public, restaurants, and food industry; intervention delivery; and project and data management for all programs. This is based on our experience in delivering research projects [24], and on systematic reviews related to nutrition improvement [25] and salt reduction [26] using mHealth technology. The structure of ASCloud is illustrated in Multimedia Appendix 1.

**Table 1:** Key features of the six programs in Action on Salt China (ASC) at stage 1.

Programs	Purposes	Rationale/design	Coverage at stage 1	Output by the end of stage 1
Program 1: health education and promotion	To improve KAP^a^ on salt reduction in the public, restaurants, and food industry; to provide a basis for the other programs	Various types of education materials were developed to improve the KAP targeting major barriers to salt reduction and sources of salt intake using evidence-based key messages	Within study sites of the intervention arms of all 4 RCTs^b^ in 6 Chinese provinces^c^	Materials (eg, manuals, fact sheets, leaflets, stickers, public advertisements, short videos, and loudspeaker audio messages) targeted at various populations and settings
Program 2: application-based intervention study (AIS)	To achieve and sustain salt reduction in school children and their families	A cRCT^d^ to test the feasibility and effectiveness of an app-based platform (AppSalt) for salt reduction. Goal setting, self-monitoring, and self-reward are the major components [20]	54 primary schools in 3 of the 6 provinces	Finalized AppSalt platform; report on effectiveness of salt reduction as measured by repeated 24-hour urinary sodium excretion; report on feasibility from the perspective of the schools, students, and families
Program 3: home cook-based intervention study (HIS)	To support families, mainly through family cooks, to reduce salt use in home cooking	A cRCT to test the effectiveness and acceptability of a community-based intervention package. Standardized education, salt intake evaluation, individualized recommendations, and reminders are the major components of intervention based on a health belief model [21]	60 communities from the 6 provinces	Intervention package; report on effectiveness of salt reduction measured by 24-hour urinary sodium excretion; report on feasibility
Program 4: restaurant-based intervention study (RIS)	To reduce salt intake when eating out by reducing salt use by restaurant cooks	A cRCT to test the feasibility and effectiveness of a restaurant salt reduction package. Social cognitive theory [22] has been adopted to develop interventions, which include (1) a standardized environment encouraging consumers to order reduced-salt dishes, (2) reminders from waiters, and (3) training cooks to reduce salt use by 10% for all, and greater reduction per consumer requirements	192 restaurants in the 6 provinces	Restaurant intervention package; report on the effectiveness of salt reduction as measured by whole food sodium analysis for each of the restaurants’ 5 best-selling dishes; report on feasibility.
Program 5: comprehensive intervention study (CIS)	To explore the experience, barriers, facilitators, and effectiveness of delivering a comprehensive salt reduction intervention	A cRCT at the township/ street level to simulate the scale-up of the intervention clusters and to test its effectiveness. The World Health Organization conceptual framework [23] was adopted to instruct the delivery of all available interventions with close engagement of local government and different sectors.	48 towns /streets in the 6 provinces	Process evaluation report; report of effectiveness of salt reduction as measured by 24-hour urinary sodium excretion.
Program 6: prepackaged food salt reduction	To encourage and support consumers to choose prepackaged foods with lower salt content (P6.1), and to work with the food industry to reduce salt use in prepackaged foods (P6.2)	Besides setting voluntary salt targets^e^, consumers are encouraged to choose foods with less salt, and food manufacturers are persuaded to reformulate the products that are high in salt. A health belief model [21] has been used to encourage the consumers to select food with less salt, while convincing food producers that foods high in salt will negatively impact sales.	Participating consumers and food producers	The FoodSwitch^f^ app (already downloaded by more than 1 million users); a website designed to raise food manufacturers’ awareness of the high salt content of their products and display ranking by salt content in product categories; by late 2019, more than 100 products have already been reformulated to contain less salt

^a^KAP: knowledge, attitude, and practice.

^b^RCT: randomized controlled trial.

^c^The 6 provinces are Heilongjiang, Hebei, Hunan, Jiangxi, Sichuan, and Qinghai, which cover the north, south, central, east, and west part of China.

^d^cRCT: cluster randomized controlled trial.

^e^Setting incremental targets for the salt content of major contributors to salt intake (eg, sauces).

^f^FoodSwitch is a smartphone app that can provide consumers with the nutrition information of a prepackaged food product (including sodium; in China, food products are labeled with sodium rather than salt in which 1 g sodium = 2.5 g salt) and a list of similar food products for making healthier choices, especially with respect to sodium reduction.

### Program Evaluation

**Figure 3:**
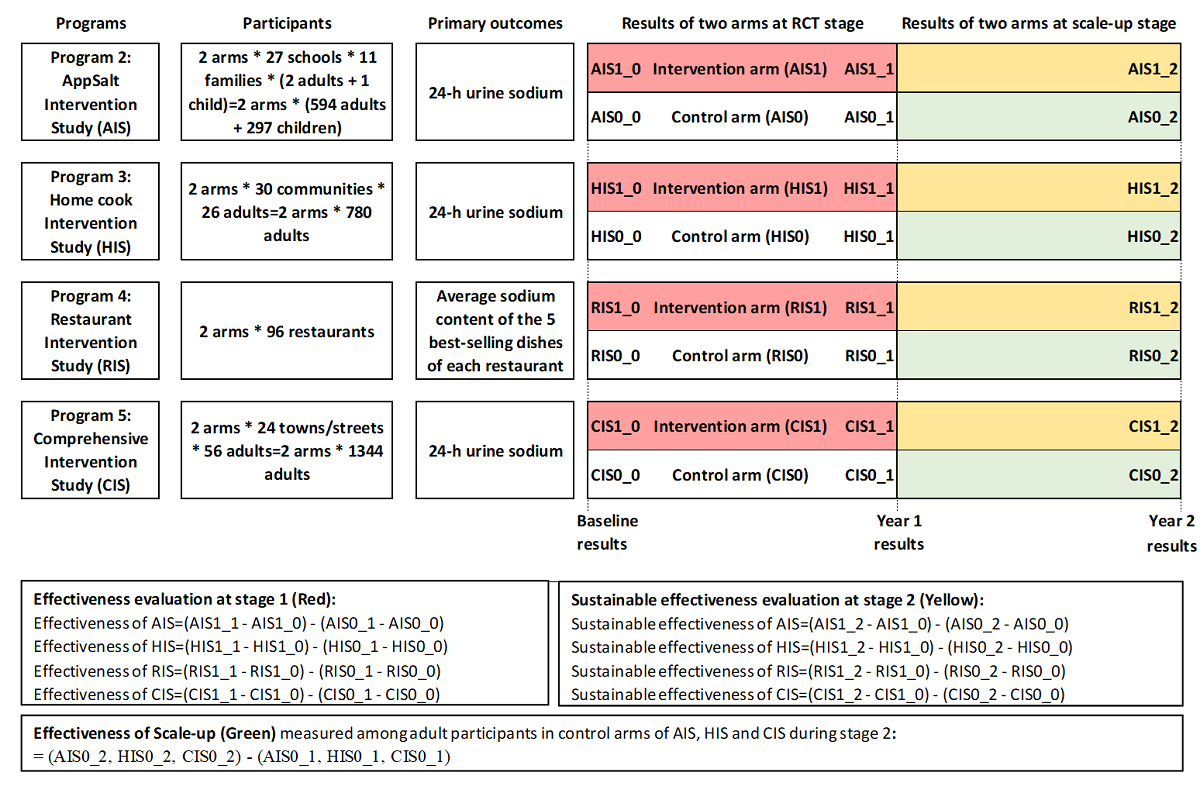
Design for the evaluation of the four cluster randomized controlled trials in Action on Salt China (ASC) Programs 2-5.

The evaluation method of the effectiveness or impact of the proposed interventions has been specifically designed for each program. In all RCTs, the 1-year effectiveness of the intervention will be assessed by comparing the salt reduction achieved between the intervention and control arms from baseline to the end of stage 1, and the sustained effectiveness over the following year will be assessed by comparing the salt reduction achieved between the intervention and control arms of AIS, HIS, and CIS from baseline to the end of stage 2. Figure 3 shows the design, sample size, and evaluation of primary outcomes for RCTs in Programs 2-5.

The sample sizes indicated in Figure 3 are equal to or slightly larger than the calculated numbers for the RCTs based on target populations (children and adults in AIS, home cooks and family members in HIS, community adults in CIS, and restaurants in RIS), primary outcomes (24-hour sodium excretion in AIS, HIS, and CIS; average sodium content of the 5 best-selling dishes for a restaurant in RIS), standard deviations of primary outcomes (85 mmol/day for 24-hour sodium excretion [19], and 1 g salt/100 g dish [27]), expected minimum salt reduction (25 mmol/day in AIS and CIS; 20 mmol/day for HIS; 0.5 g/100 g dish for RIS [27]), intraclass correlation coefficient (0.05 for AIS, HIS, and CIS [19], and no cluster effect for RIS), drop-out rate (<20%) for individuals/clusters, type I error (5%), and power (>80%), as well as reasonable cluster size (11 students and families in each school in AIS, 13 families in each village in HIS, and 30 adults in each village in CIS).

Secondary outcomes will consist of process evaluation, changes in KAP on salt intake, and economic evaluation. Overall KAP on salt, and overall salt intake levels will also be estimated by pooling the data collected in AIS, HIS, and CIS at baseline, at the end of stage 1, and at the end of stage 2.

Statisticians will be blinded to the intervention assignments during data analysis. An intention-to-treat approach will be adopted for analysis of the primary outcomes. The effect of the intervention on the outcomes will be tested using linear mixed models. For AIS, HIS, and CIS, participants will be nested within family units and families nested within clusters (schools, villages, or towns). Group (intervention, control), time (baseline, end of trial), and time-by-group interaction will be included in the model, with the interaction effect indicating differential change according to group from baseline to the end of the trial. We will adjust for the stratification and potential confounding variables at randomization.

### Data Collection and Management

Our study will have three major data outputs: (1) data obtained from the evaluation of each RCT’s effectiveness (Table 2); (2) data obtained from the process monitoring and evaluation, which will consist of quantitative data automatically generated by the smartphone apps used as intervention tools in the RCTs, as well as qualitative data collected for process evaluation; and (3) monitoring and evaluation data on the coverage and usage of the salt reduction materials and tools, which will be recorded by ASCloud during the scaling-up phase. With the exception of the routine work log and qualitative data collected for process evaluation, most of the data will be collected using specially designed electronic systems, including a mobile device-based electronic data capture system (mEDC) and the ASCloud server, which can capture activities such as log in/log out and access to certain features through different kinds of front ends, Web portals, and mobile apps. The mEDC has an improved process and quality control feature compared with a traditional EDC, and has been validated and widely used in other clinical trials [24,28].

All cleaned and locked datasets for each RCT, together with the study design, questionnaires, code lists, and definitions of databases and variables, will be stored in TGI China, with a unique ID number attached but no personal identifiers, following an established standard operating procedure for data security. To guarantee data security, the mobile app developer (ie, the Information Technology team at Beihang University) will follow the “Mobile Application Information Service Regulation” issued by the Cyberspace Administration of China in 2016 [30]. Although personal data will be accessible to the app developer, disclosure of such information is prohibited.

**Table 2:** Data collection in the four randomized controlled trials in Action on Salt China (ASC) Programs 2-5.

Questionnaires		Baseline				Year 1 and 2 follow-up
		AIS^a^	HIS^b^	RIS^c,d^	CIS^e^	
**Demographics**						
	Sex	✓	✓	✓	✓	—^f^
	Age	✓	✓	✓	✓	—
	Education	✓	✓	✓	✓	—
	Marriage	✓	✓	✓	✓	—
	Income	✓	✓	✓	✓	—
	Medical Insurance	✓	✓	✓	✓	—
**Knowledge, attitude, and practice (KAP)**						
	Preference for salt	✓	✓	✓	✓	As baseline
	Awareness of salt recommendation	✓	✓	✓	✓	As baseline
	Awareness of salt and hypertension	✓	✓	✓	✓	As baseline
	Awareness of low-sodium salt	✓	✓	✓	✓	As baseline
	Awareness of salt labeling	✓	✓	✓	✓	As baseline
	Attitude to low-salt diet	✓	✓	✓	✓	As baseline
	Attitude to low-salt behavior	✓	✓	✓	✓	As baseline
	Salt use during cooking	✓	✓	✓	✓	As baseline
	Frequency of eating out	✓	✓	✓	✓	As baseline
	Ordering dishes with reduced salt when eating out	✓	✓	✓	✓	As baseline
**Lifestyle**						
	Smoking	✓	✓	—	✓	As baseline
	Drinking	✓	✓	—	✓	As baseline
	Physical activity	✓	✓	—	✓	As baseline
**Disease history**						
	Hypertension	✓	✓	—	✓	As baseline
	Anti-hypertensive medication use	✓	✓	—	✓	As baseline
	Other chronic diseases	✓	✓	—	✓	As baseline
**Physical examination**						
	Height	✓	✓	—	✓	As baseline^g^
	Weight	✓	✓	—	✓	As baseline
	Waist circumference	✓	✓	—	✓	As baseline
	Blood pressure	✓	✓	—	✓	As baseline
	Heart rate	✓	✓	—	✓	As baseline
**24-hour urinary excretion^h^**						
	Sodium	✓	✓	—	✓	As baseline
	Potassium	✓	✓	—	✓	As baseline
	Creatinine	✓	✓	—	✓	As baseline
	Albumin	✓	✓	—	✓	As baseline
	Calcium	✓	✓	—	✓	As baseline
**Restaurants**						
	Salt-specific environmental factors	—	—	✓	—	As baseline
	Recipe of the 50 best-selling dishes	—	—	✓	—	—
	Percentage of consumers who choose lower salt foods	—	—	✓	—	—
	Usage of salt and highly salted foods	—	—	✓	—	As baseline
	Provision of salt reduction services	—	—	✓	—	As baseline
**Whole food laboratory test**						
	Sodium content of the 5 best-selling dishes	—	—	✓	—	As baseline

^a^AIS: app-based intervention study.

^b^HIS: home cook-based intervention study.

^c^RIS: restaurant-based intervention study.

^d^The primary outcome of RIS is the change of salt use among the study restaurants measured by whole food sodium analysis for the 5 best-selling dishes in each restaurant. Twenty consumers will be invited to take part in a simple survey at baseline and at the end of 2 follow-ups.

^e^CIS: comprehensive intervention study.

^f^Not applicable.

^g^Only the height of children in AIS will be measured during the follow-up visit at year 1 and year 2.

^h^The quality control for 24-hour urine collection refers to the protocol of AIS [29].

### Patient and Public Involvement

During development of the overall design of ASC and the specific protocols for each RCT, people from the target populations and those involved in the implementation of the interventions were consulted at least once through meetings, teleconference, and site visits. The consulted individuals included primary school teachers, parents of primary school children, family cooks, consumers of prepackaged food, food producers, restaurant staff, community residents, and policy makers. All ideas on new interventions, opinions on the feasibility of specific interventions, and suggestions to improve their design and implementation were carefully considered. All participants will be informed of the study progress by regular communication via the ASC newsletter, WeChat, and website. Upon completion of the study, we will disseminate the results to all participants and discuss the translation of our study findings to practice.

## Results

The duration of ASC is from June 1, 2017 to March 31, 2021, with March 31, 2020 as the split point for stage 1 and stage 2. The preparation of the 4 RCTs and their baseline investigations were completed at the end of March 2019. Protocols of the intervention packages or intervention components that proved to be effective at stage 1 will be made available and scaled up by combining them into an existing national initiative such as Healthy Lifestyle Campaign for All [31] for stage 2. The status of all ASC programs is summarized in Textbox 1.

All trials have been approved by Queen Mary Research Ethics Committee in the United Kingdom (QMERC2018/13 for AIS, QMERC2018/15 for HIS, QMERC2018/16 for CIS, and QMERC2018/14 for RIS) and the Institutional Review Boards of Peking University (IRB00001052-18051 for AIS), Chinese CDC (No. 201801 for HIS), National Center for Chronic and Noncommunicable Disease Control and Prevention, China CDC (No. 201807 for CIS), and National Institute for Nutrition and Health, China CDC (20180314 for RIS). Written informed consent forms have been obtained from all participants according to well-established practices. For children, participant assent and parental written consent have been obtained. All participants are free to discontinue their participation at any time with no explanation required.

By the end of 2019, three steering committee meetings have been convened. Nine presentations have been made in international (four) and national (five) meetings. The Chinese National Health Commission, especially the divisions of disease control, food safety, and health education, is looking forward to adopting the evidence-based salt reduction packages in ASC in 2020, which could help support the Healthy China Initiatives launched in July 2019 [33].

**Status of the programs of Action on Salt China (ASC).**

- Program 1: Health education and promotion
  - KAP (knowledge, attitude, and practice) questionnaire: pilot tested and now in use by all RCTs (randomized controlled trial).
  - Education materials: 1 manual, 3 leaflets, 2 public advertisements (15 s and 30 s), 3 short videos targeting 3 major settings for salt intake (home cooking, eating out, and groceries), 8 loudspeaker audio messages for rural village use, and several others; ready for use in RCTs.
- Programs 2-5: app-based intervention study (AIS), home cook-based intervention study (HIS), restaurant-based intervention study (RIS), comprehensive intervention study (CIS)
  - Intervention package development for all 4 RCTs: completed.
  - Participant recruitment: 592 children and 1184 adults recruited for AIS, 1576 home cooks recruited for HIS, 2694 residents recruited for CIS, and 192 restaurants recruited for RIS.
  - Baseline surveys: completed, including 24-hour urine collection in AIS, HIS, and CIS, and whole food sodium analysis for the 5 best-selling dishes of each participating restaurant in RIS.
  - Randomization: Schools/communities/restaurants randomly assigned to intervention and control arms after completion of baseline surveys.
  - Intervention implementation: Ongoing
  - RCT follow-up assessment: completed by the end of January 2020 for AIS, HIS, and CIS, and will be completed by the end of May 2020 for RIS.
- Program 6: Prepackaged food salt reduction
  - FoodSwitch was released June 16, 2019 and has been downloaded by over 10,000 users as of November 2019. It can be found by searching “Shi Xian Zhi” in most app markets and WeChat (a popular communication app in China), and can be used by consumers to choose lower-salt prepackaged foods. Shi Xian Zhi is Chinese pinyin, with Shi indicating food, and Xian Zhi for prophet.
  - ASC’s official website now provides a purpose-built page for food producers, based on the data collected through FoodSwitch.
- The information system (ASCloud)
  - ASC’s official website [32] was launched in September 2018. To avoid contamination of the RCTs’ control arms, it will remain open mainly for internal use and for food producers during stage 1.
  - Several smartphone apps and WeChat applets (ie, very small apps) have also been developed to help consumers choose foods with less salt (FoodSwitch, Shi Xian Zhi in Chinese pinyin), to help families estimate their salt intake (KnowSalt, Jia Ting Yong Yan Ce Liang in Chinese pinyin, used in HIS and CIS), to help deliver a series of health education and activities on salt reduction in schools for the school children and their families (AppSalt, Jian Yan, used in AIS), and to help project implementation and quality control (one app per RCT). Jian Yan is Chinese pinyin, with Jian meaning health and Yan meaning salt.
- Laboratory tests
  - One top-level central laboratory has been contracted for the assay of all urine samples collected in AIS, HIS, and CIS, and the whole food sodium analysis in RIS.

## Discussion

### Expected Outputs and Potential Impact

ASC is a research unit led by a strong multidisciplinary team with members from international and national institutes engaging in nearly all relevant areas on salt reduction in China. The ASC program is comprehensive, including a salt awareness campaign, legislation support, and RCTs evaluating the strategies dealing with key challenges on salt reduction in China. ASC uses innovative digital health technologies to support the delivery of interventions as well as project and data management. The real-time monitoring and process evaluation can help to increase the fidelity of complex interventions.

As a unit, the six programs of ASC will provide a set of novel approaches to reduce salt intake in China. The expected outputs of ASC include: (1) several evidence-based intervention packages addressing major sources of salt intake; (2) evidence-based salt reduction strategies and experience on policy advocacy along with scaling-up in different regions and populations; and (3) study reports and publications to highlight the gaps, needs, barriers and facilitators, and strategies in salt reduction among different populations. All these major outputs will make significant contributions to the national policies, programs, and initiatives on the prevention and control of noncommunicable diseases as well as the promotion of healthy diets and healthy lifestyle in China. Owing to the strong policy support, multidisciplinary research team, and close partnership with all key national agencies, ASC is most likely to succeed in achieving its intended objectives.

To date, ASC has already made significant progress in achieving its academic impact. As shown in Textbox 1, ASC has attracted the attention of key departments of the Chinese central government. With the support of relevant government agencies, the development of detailed scaling-up plans is underway. If the program is implemented and sustained across China, it will reduce population salt intake and thereby prevent hundreds of thousands of strokes, heart attacks, and heart failure each year, leading to major cost-savings to individuals, their families, and the health service. Although our study will be carried out in China, the outcomes could potentially be adopted by many other countries. Additionally, our model on salt reduction could possibly be adapted for other dietary and lifestyle changes to prevent CVD and other noncommunicable diseases, which will have major public health implications.

### Future Dissemination

The findings of this study will be disseminated widely through conference presentations, peer-reviewed publications, press releases, and social media. In China, the results and effective interventions will be disseminated nationwide through the existing system of health education, disease control, and prevention. Furthermore, the results will be disseminated worldwide through World Action on Salt and Health [34], which is a global nonprofit organization of 600 members from 100 countries with the mission to improve the health of populations by reducing salt intake.

### Potential Limitations and Risks

The interventions for each program of ASC are complex. Low compliance to the intervention may lead to negative results for the primary outcomes. In addition, the implementation of collecting 24-hour urine is very demanding. Fidelity to interventions and quality control for effectiveness evaluation are both critical to the success of the programs. With the widespread implementation of Healthy China 2030 initiatives, salt reduction is being promoted by many other programs. Therefore, it might be very difficult to distinguish the contributions of ASC from those of other programs.

### Conclusion

The ASC project is progressing smoothly. The intervention packages and components evaluated at stage 1 will provide strong support for salt reduction in China, and could potentially be adopted by many other countries worldwide.

## Appendix

Multimedia Appendix 1 Structure of Action on Salt (ASC) information system (ASCloud) developed by Beihang University. AIS: app-based intervention study; HIS: home cook-based intervention study; RIS: restaurant-based intervention study; CIS: comprehensive intervention study; mEDC: mobile phone app based on an electronic data collection system; PM: project management; MS: management system; NINH: National Institute for Nutrition and Health, China CDC; TGI: The George Institute for Global Health; NCD Division: Division of NCD Control and Community Health, China CDC; NCD Center: National Center for Chronic and Non-communicable Disease Control and Prevention, China CDC; CFSA: China National Center for Food Safety Risk Assessment.

Multimedia Appendix 2 Peer review comments and responses from National Institute for Health Research.

Multimedia Appendix 3 CONSORT-eHEALTH checklist (V 1.6.1).

## Abbreviations

AIS: app-based intervention study
ASC: Action on Salt China
CCHE: Chinese Center for Health Education
CFSA: China National Center for Food Safety Risk Assessment
China CDC: Chinese Center for Disease Control and Prevention
CIS: comprehensive intervention study
CVD: cardiovascular disease
HIS: home cook-based intervention study
KAP: knowledge, attitude, and practice
mEDC: mobile device-based electronic data capture system
NIHR: National Institute for Health Research
RCT: randomized controlled trial
RIS: restaurant-based intervention study
SIPS: scale-up intervention package on salt reduction
TGI: The George Institute
WHO: World Health Organization
