# Supplementary figures and images for: Reducing Salt Intake in China with “Action on Salt China” (ASC): Protocol for Campaigns and Randomized Controlled Trials
Source: JMIR Res Protoc. 2020 Apr 9;9(4):e15933. doi: 10.2196/15933 (PMC7180507; doi:10.2196/15933)

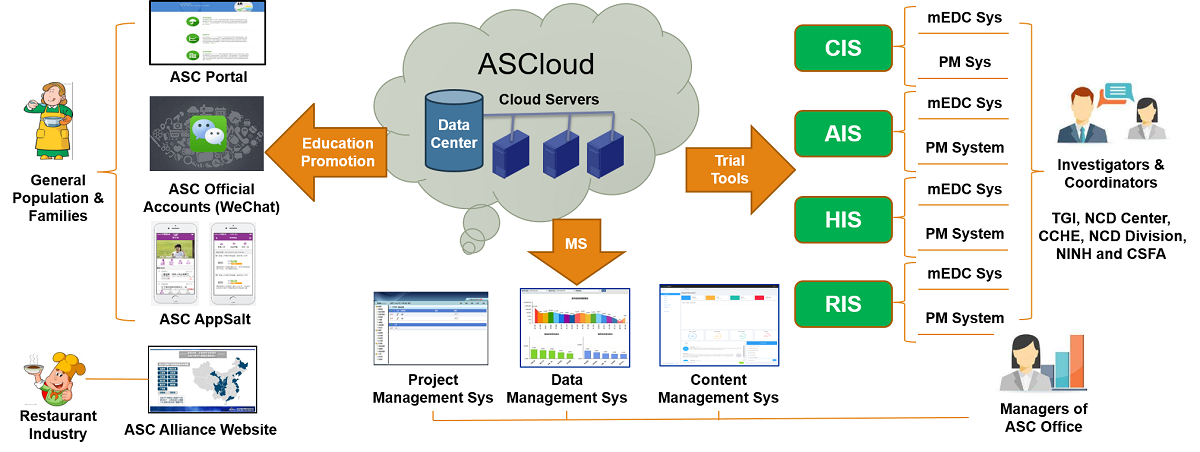

Supplement: Multimedia Appendix 1 [file resprot_v9i4e15933_app1.png]
