# Supplementary material for: Reducing Salt Intake in China with “Action on Salt China” (ASC): Protocol for Campaigns and Randomized Controlled Trials
Source: JMIR Res Protoc. 2020 Apr 9;9(4):e15933. doi: 10.2196/15933 (PMC7180507; doi:10.2196/15933)
Supplement: Multimedia Appendix 2 [file resprot_v9i4e15933_app2.pdf]

National Institute for
Health Research

RESEARCH PROPOSAL EXTERNAL REVIEW OPEN COMMENTS

Review Questions

Question and Response
Comments on importance of the proposed research area<br><br>High salt intake is very common in Asian countries diet, so a program that properly target this behaviour could reduce the health risk from such dietary habit. As this program target the most basic behaviour enjoyed across all socioeconomic strata, this could reduce health inequalities in the specific country.<br>There is a clear plan to strengthen the capacity in developing countries through studentship, fellowship and studentship.<br>Even though the application managed to identify the gap to establish the importance of reducing salt, it does not managed to point out why they feel the existing local program in China is not as comprehensive/successful compared to the planned program.
Comments on the deliverability of the proposed programme of research<br><br>The proposal appears to consist of good mix of researchers from various discipline, academician and policy makers. If there is an area to be improved, then perhaps the researchers can consider to have a formal food industry/operator partner to establish the practicality of the intervention.<br>The proposal has detailed the role and relationship for the various organizations involved in the study. The fund requested seems reasonable.
Comments on the overall quality of the application<br><br>Well thought application with good capacity building planning.
Other information
Do you have any questions for the applicants that you would like the opportunity for the applicants to respond to prior to the proposal being considered by the funding board?

National Institute for Health Research

RESEARCH PROPOSAL EXTERNAL REVIEW OPEN COMMENTS

Review Questions

Question and Response
Comments on importance of the proposed research area<br><br>The area of salt reduction has immense public health value especially in a country like China where the salt intake is quite high. The focus of the research by working with researchers and policy makers in China, the UK team has ensured that the research can be implemented and sustain. The topic also focuses on the preventive approach rather than managing the disease burden due to high salt intake.
Comments on the deliverability of the proposed programme of research<br><br>The research team has ensured that the team members in China have both researchers and policy experts so as to ensure that the research can be implemented well. The team also gone to various lengths to ensure that the research implementation feasibility through right partnerships with relevant personnel based in China.
Comments on the overall quality of the application<br><br>The application quality is quite good, and provides all details needed for ensuring the research implementation challenges in China. The details of the research team is detailed and ensures that the reviewer understands their importance and capability for the research implementation in China.
Other information<br><br>None.
Do you have any questions for the applicants that you would like the opportunity for the applicants to respond to prior to the proposal being considered by the funding board?<br><br>Does the research focus on documenting the implementation costs? Also, is there a focus to conduct a cost-benefit analysis?

Applicant Response To External Review

Applicant Response

We are very grateful to both reviewers for reviewing our application. We appreciate their very helpful comments. We have answered all questions in detail below.

Question 1: Even though the application managed to identify the gap to establish the importance of reducing salt, it does not manage to point out why they feel the existing local program in China is not as comprehensive/successful compared to the planned program.

Answers to Question 1

We agree this is an important point. Due to the word limit, we were unable to describe this in detail in the original application. Salt reduction has been included in the promotion of healthy lifestyle which is a key priority of China's health development agenda for the prevention and control of non-communicable diseases (NCDs). The government has released a few relevant documents ("Healthy China 2030" plan aiming to build a healthier China through comprehensive efforts, The 13th Five Year Plan for Health, and China Mid-term and Long-term Plan for NCD Prevention and Control (2017-2025)). Some of these plans have been or are being implemented in China. However, all of these programmes have focused on promoting overall healthy lifestyle and there is no detailed salt reduction strategy and, as a consequence, the impact of such programmes on reducing population salt intake is doubtful.

In the following section, we will briefly describe the existing programmes and outline how our proposed programmes have higher likelihood of success in achieving a sustainable reduction in population salt consumption in China.

Currently there are three main programmes that have either included salt reduction as a component or focused on salt reduction.

(1) China Healthy Lifestyle for All Initiative. This is a government-led initiative with a focus on promoting healthy behaviors which includes reducing fat, salt and sugar intake as well as promoting dental health, body weight control and bone health. The salt reduction component in this initiative is mainly done by general education. There is lack of detail on salt reduction strategy, for example, how to reduce salt from different sources in the diet, i.e. food prepared at home, pre-packaged food and restaurant food. In addition, there is lack of specific targets of salt reduction. Furthermore, there is no monitoring programme or evaluation for salt reduction either at individual/family level or population level.

(2) Demonstrational Areas for Comprehensive NCD Prevention and Control Initiative. This initiative is led by the NCD center of China CDC aiming to develop a comprehensive system to monitor and manage NCDs. The initiative emphasises the leadership of the local government and is primarily operated by the local CDCs and community health service centers. "Healthy lifestyle" is also one component of this initiative using the same materials developed in "China Healthy Lifestyle for All Initiative" without effective evaluation. Salt reduction is a vague component. Furthermore, the local CDCs and community health service centers do not have adequate capability to develop salt reduction measures by themselves.

Applicant Response

(3) Shandong-Ministry of Health Action on Salt Reduction and Hypertension (SMASH). This programme has been jointly carried out by China Ministry of Health and Shandong provincial government in Shandong province since 2011. We acknowledge that it is a relatively comprehensive salt reduction programme that involves various stakeholders including schools, food industry, health providers, etc. Data analysis of SMASH has not yet completed and the effectiveness of this programme is unknown. However, there are some known major drawbacks including: 1) less involvement of end beneficiaries, i.e. the general public. This is partially due to a lack of evidence-based tools and methods, e.g. FoodSwitch, KnowSalt and School-EduSalt, to empower the individuals to know their salt intake, sources of salt in their diet, salt targets or progress made; 2) less involvement of some relevant national agencies, e.g. Chinese Center for Health Education and National Institute for Nutrition and Health, and some important social or private stakeholders/operators, e.g. China Cuisine Association, China National Food Industry association, supermarket chains and network catering platforms; 3) lack of strategies or standards of salt reduction (e.g. salt target setting) for pre-packaged foods, resulting in low participation from food manufacturers; and 4) no consideration of facilitating salt reduction by improving the nutrition information panel (contents, types and formats) through the China National Center for Food Safety and Risk Assessment. Taken together, SMASH is a pilot study in one province with various limitations, it is unknown whether SMASH can be adopted by other provinces and currently there is no clear plan for scaling up.

In view of the above, our proposed Unit, Action on Salt China (ASC), aims to develop and implement a comprehensive and effective national salt reduction programme by taking advantage of the substantial expertise and experience from the UK and China, to overcome the problems and challenges in the existing programmes. We have taken the following strategies to ensure our success.

First, we have gathered a strong team. Two members have been directly involved in the existing programmes, Dr. Jing Wu is the Director of NCD Control and Community Health Division, China CDC, which is the operation center of the China Healthy Lifestyle for All Initiative, and Prof. Jixiang Ma, Deputy Director of NCD center, China CDC who is leading the Demonstrational Areas for Comprehensive NCD Prevention and Control Initiative, and the SMASH programme. Researchers and officers from the George Institute for Global Health China, Chinese Center for Health Education, National Institute for Nutrition and Health as well as the China National Center for Food Safety and Risk Assessment, have joined us to support the implementation of ASC.

Furthermore, our UK team have extensive experience in population salt reduction in both developed and developing countries, and have helped many countries to develop a salt reduction strategy. Taken together, our strong team of leading experts with substantial experience on salt reduction both in China, UK and worldwide will ensure our proposed programmes are successful.

Second, we have developed an evidence-based, practical and effective strategic plan. With the lessons learned from previous work in China, our application has proposed several programmes to target various sources of salt in the Chinese diet and have set specific and measurable short-term, medium-term and long-term objectives for each programme with rigorous evaluation plan. Most of our proposed programmes will have an impact across the whole country, e.g. salt awareness education, salt target setting for pre-packaged foods and nutrition labelling. In China, there is a rapid increase in the consumption of pre-packaged food, and therefore it is important to reduce salt content in these types of food as well. The UK's successful salt reduction programme has clearly demonstrated that a target-based approach is effective in reducing the salt content in processed food [Brinsden et al. BMJ Open 2013;3:e002936; Hashem et al. BMJ Open 2014;4:e005051]. We will adopt this approach by setting incremental salt reduction targets for different categories of pre-packaged foods. Moreover, we will work on improving nutrition labelling regulation. This will have fundamental impact on the nutrition labeling practice of the food industry and will encourage food manufacturers to reformulate their food

Applicant Response
composition, as well as helping the consumers to choose lower-salt food. Furthermore, we have proposed to develop and provide tools (KnowSalt and FoodSwitch) to empower the general public to choose healthier foods and set salt reduction targets and monitor progress.<br><br>Third, we have proposed comprehensive capacity building activities. We will conduct various training activities to enhance the capacity of all stakeholders including policy makers and local health workers to ensure the programmes are implemented successfully. In addition, fellowship, studentship, monthly research trainings and annual salt reduction conference will help the researchers in China improve their capacity.<br><br>In summary, the existing local or national programmes are not adequate to address the high salt intake problem in China, although they provide valuable resources and experience. Due to the lack of effective salt reduction programmes, hundreds of thousands of people suffer or die from stroke and heart disease unnecessarily each year. Our proposed ASC is therefore timely. By learning from both international and national experience, we have formed a strong team to develop and implement an evidence-based, practical and effective strategy to reduce salt intake in China. As our programmes target changes at national policies, there is a high likelihood of success, and more importantly the programme will be sustainable to achieve gradual stepwise progressive reductions in salt intake.<br><br>Question 2: If there is an area to be improved, then perhaps the researchers can consider to have a formal food industry/operator partner to establish the practicality of the intervention.<br><br>Answers to Question 2<br><br>The food industry plays an important role in salt reduction especially in reducing salt from pre-packaged food and sauces such as soy sauce which is very high in salt and commonly used in the Chinese cooking. Engagement with the food industry is one of the key elements of our proposed programme. Although our team did not include an individual from the food industry as a co-investigator, two of our team members have had many years' experience of working closely with the food industry in China.<br><br>As a key stakeholder, the food industry will be involved in various aspects of our proposed programmes.<br><br>First, we will work closely with food producers and food providers on reducing salt from pre-packaged food including sauces e.g. soy sauce and from eating out of home. Our team members in China, Professor Bing Zhang from National Institute for Nutrition and Health of China CDC and Professor Junhua Han from National Center for Food Safety Risk Assessment, have worked with leading food industries such as China National Cereals, Oils and Foodstuffs Corporation (COFCO), China National Salt Industry Corporation, PepsiCo and Group Danone. We will mobilize the participation of all major food producers and providers through China Cuisine Association and China National Food Industry Association. Additionally, we will work with large-scale chain supermarkets and retailers (Carrefour, Walmart, Wumart, MerryMart, etc.) as well as network catering platforms such as Baidu take-away to encourage the provision of lower-salt food.<br><br>Second, to reduce salt from pre-packaged food, we will set incremental lower salt targets for different categories of foods with a clear timeframe for the food industry to achieve. The UK's successful salt reduction model [He et al. J Hum Hypertens 2014;28:345] will be adapted and, in particular, tailored according to the Chinese dietary pattern. The target setting will be done based on salt content in food from our FoodSwitch database (already collected over 25,000 products) and the contribution of different food categories to total salt consumption from national dietary survey. It is important to

Applicant Response
involve key stakeholders, particularly the food industry to ensure the targets are practical and achievable. The effect will be evaluated through continuous regular update of FoodSwitch database. Our team member, Prof. Puhong Zhang at the George Institute is currently leading the FoodSwitch project in China and Prof. Bing Zhang from National Institute for Nutrition and Health of China CDC is leading the national dietary survey. We will work closely with the food industry to ensure they meet the targets. To further encourage food manufacturers to take action, we will adopt "name and shame" approach via various media channels, i.e. appraise the manufacturers that have made progress in reducing salt and shame those who do not take action. This approach has been proven effective as demonstrated by the UK's successful salt reduction programme.<br><br>Third, food industry is also a key stakeholder in the amendment of nutrition labelling regulations, which will be undertaken by National Center for Food Safety Risk Assessment (NFSA). This work will be led by our team member Dr. Junhua Han who is the director of Division I of Applied Nutrition, NFSA. Barriers and facilitators of the nutrition labelling will be investigated not only from the perspective of consumers but also from the point of food manufacturers to ensure the practicality of the amendment. Moreover, through the participation, the food industry will understand that salt reduction is in line with their long-term development and will take active action to reduce salt.<br><br>Question 3: Does the research focus on documenting the implementation costs? Also, is there a focus to conduct a cost-benefit analysis?<br><br>Answers to Question 3<br><br>Yes, we will have detailed documentation of all activities undertaken and their associated costs to enable us to conduct health economic evaluation (HEE).<br><br>HEE is essential for central and local governments to set priorities for activities on salt reduction. We plan to conduct economic evaluations for ASC as a whole and also for each specific project embedded in the programmes. At the initial stage (year 1), the overall HEE plan and plans for each project will be finalised together with study protocols, including study participants, interventions, outcomes, sample size, types of costs, data collection methods, data analysis plan and communication plan. The perspectives to be adopted in all studies will be societal, to account for the inter-sectoral nature of the intervention (including health, education and industry).<br><br>The implementation costs incurred in each of the projects delivering the interventions and the methods used will conform to published guidelines (Conteh and Walker, Health Policy Plan. 2004;19:127-35). Costs to be documented will include costs in cash and in kind, direct and indirect, in establishing and running the interventions, e.g. the costs of training, development of materials and tools, education, coordination as well as intervention implementation. The costs of these resources, in particular staff times devoted to these activities, will be based on interview with staff from the projects and partner organisations and extracts from financial statements of these organisations and project budgets. Adjustment will be made to exclude protocol driven costs associated with conducting research such as research protocol development, effectiveness evaluation and publication.<br><br>The reductions in salt intake and systolic blood pressure (SBP) will be used as the indicators of effectiveness (cost-effectiveness analysis). Based on specific study design (randomised controlled trial and/or before-after health intervention), cost-effectiveness ratios, i.e. incremental cost of achieving a 1 gram reduction in salt intake and the cost of achieving a 1 mmHg reduction in SBP, will be calculated for all of the projects developed by ASC. These will allow us and policy makers to find the most cost-effective strategies or methods for salt reduction.

**Applicant Response**
Whilst these project-based findings indicate the costs relative to outcomes achieved within the duration of the projects, they do not address the question of how such investment influences long term costs and outcomes in terms of survival, disability and downstream costs to the health system and the community. To do this, Markov models will be developed to extrapolate these project-based cost-effectiveness findings into estimates of the incremental cost per life year saved and incremental cost per Disability Adjusted Life Year (DALY) averted (cost-utility analysis. Briggs et al. Decision Modelling for Health Economic Evaluation. Oxford: Oxford University Press, 2006). This will be done by projecting the long term impact of reductions in dietary salt and SBP on cardiovascular disease (CVD) events. The models will be based on annual cycles in which the cohort of individuals with and without the interventions will be assessed separately. Using evidence from the literature we will assess their annual transition, initially from post trial health states and then in subsequent years between each of the specified Markov health states (e.g. full health, CVD event, death). The annual probabilities of transition between states, disability and health care costs associated with each will be derived by review of the literature. This aspect of evidence review and modelling will be important as effects on survival and disability are not expected within the duration of the projects and it is essentially in these long term costs and outcomes in which we would expect the interventions to be of benefit. The model will extrapolate a 80 year time horizon (or longer if needed) in which the entire cohort are expected to have died.<br><br>With appropriate discounting, the average per individual cost, disability and survival from each cohort (with and without intervention) over a lifetime will fold out of the model and the differences used to estimate lifetime incremental cost-effectiveness/utility of the intervention strategy.<br><br>The modelling will incorporate extensive sensitivity analyses in which parameters where there is uncertainty (e.g. sustained lower salt intake beyond the programme, costing assumptions, discount rate) will be varied. These findings will enable the cost-effectiveness of the intervention tested in this study to be benchmarked against standard criteria such as that of the WHO of an incremental cost per DALY averted of less than three times gross national income.<br><br>Based on the model-based projection, the future cost saved due to reduced service, medication, hospitalisation and early death as a result of salt/blood pressure reduction will be used to compare with the implementation costs directly to see if the interventions or ASC as a whole is cost saving (cost-benefit analysis). The unit cost for all relevant service, medication and hospitalisation will be based on existing literature or newly collected data if no existing data are available. Sensitivity analysis mentioned above will also be considered in the cost-benefit analysis.<br><br>Our team, both in the UK and China, have the expertise in HEE. In China, the George Institute for Global Health has a HEE team who have the experience in performing HEE in health promotion, especially on salt reduction programme such as School-EduSalt. From our UK team, Dr. Francesca Cornaglia is an expert in HEE.
